# Supplementary figures and images for: A 10 years update of effects of exercise on depression disorders—in otherwise healthy adults: A systematic review of meta-analyses and neurobiological mechanisms
Source: PLoS One. 2025 May 5;20(5):e0317610. doi: 10.1371/journal.pone.0317610 (PMC12052119; doi:10.1371/journal.pone.0317610)

S1 Fig. Funnel Plot Adjusted with Trim-and-Fill Method


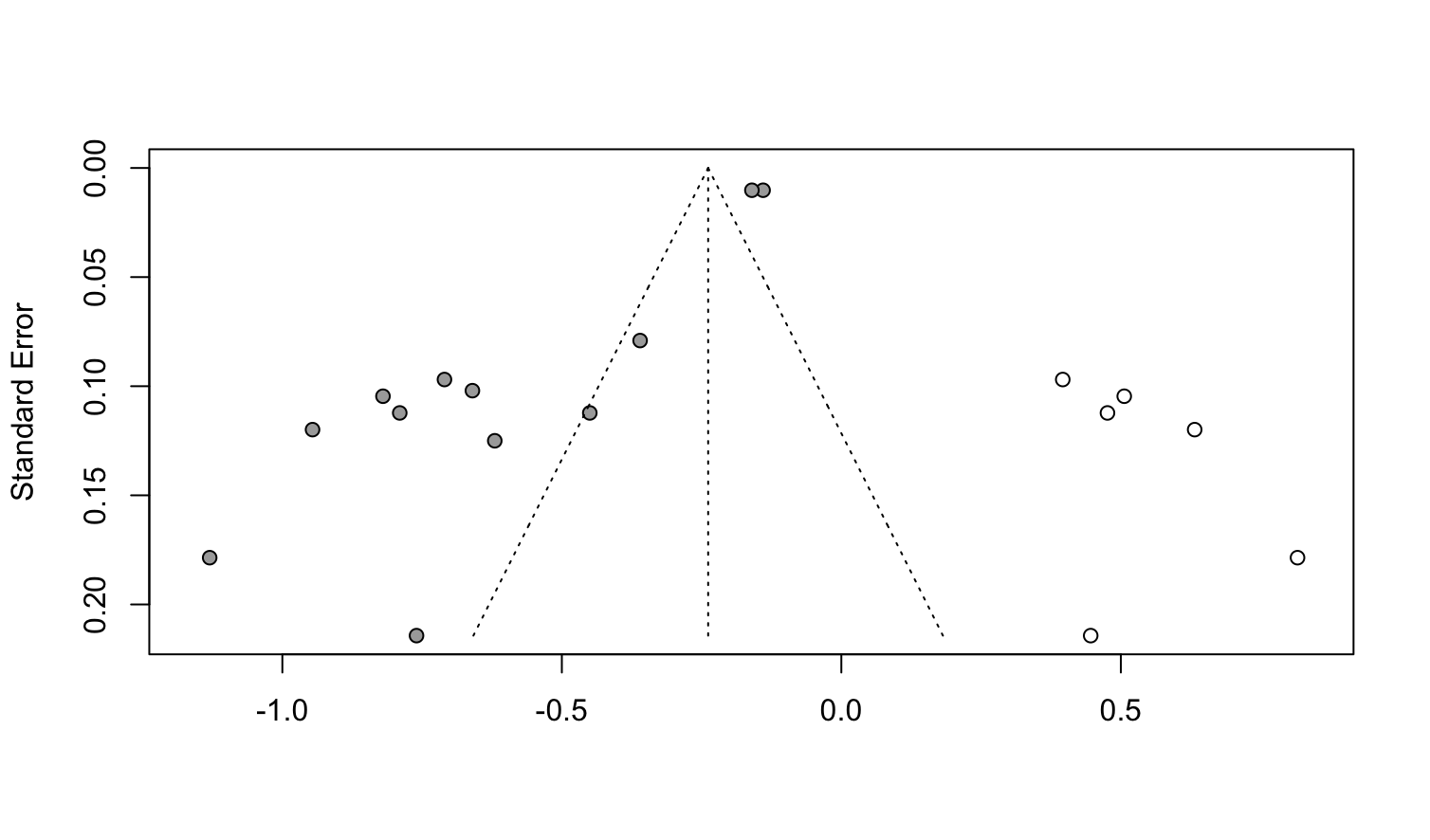

Supplement: S1 Fig — This figure shows the adjusted analysis of publication bias. (DOCX) [file pone.0317610.s001.docx]

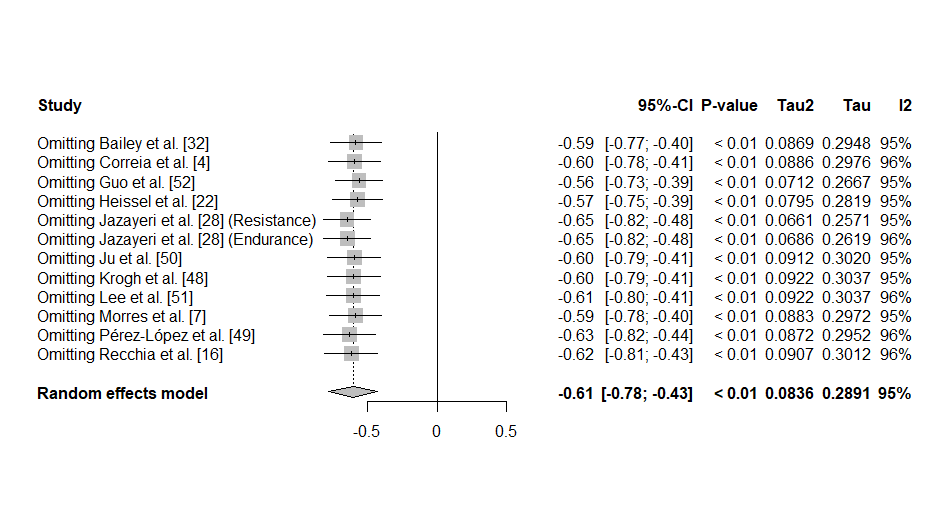


*S2 Fig. Forest Plot of Leave-One-Out Sensitivity Analysis*

Supplement: S2 Fig — The graph shows the impact of excluding individual studies on the overall effect. (DOCX) [file pone.0317610.s002.docx]
