# Supplementary material for: A 10 years update of effects of exercise on depression disorders—in otherwise healthy adults: A systematic review of meta-analyses and neurobiological mechanisms
Source: PLoS One. 2025 May 5;20(5):e0317610. doi: 10.1371/journal.pone.0317610 (PMC12052119; doi:10.1371/journal.pone.0317610)
Supplement: S1 Table — This table lists studies that were excluded during the selection process, including the exclusion criteria. (DOCX) [file pone.0317610.s003.docx]

**S1 Table. List of Excluded Studies**

| **Study** | **Reference** | **Reason for Exclusion** |
| --- | --- | --- |
| 1. Pérez Bedoya É et al.,   2023 | Pérez Bedoya É A, Puerta-López LF, López Galvis DA, Rojas Jaimes DA, Moreira OC. Physical exercise and major depressive disorder in adults: systematic review and meta-analysis. Sci Rep. 2023;13(1):13223. | Comorbid diseases |
| 1. Singh et al., 2023 | Singh B, Olds T, Curtis R, Dumuid D, Virgara R, Watson A, et al. Effectiveness of physical activity interventions for improving depression, anxiety and distress: an overview of systematic reviews. Br J Sports Med. 2023;57(18):1203-9. | Comorbid diseases (cancer) |
| 1. Sun et al., 2023 | Sun M, Liu C, Lu Y, Zhu F, Li H, Lu Q. Effects of Physical Activity on Quality of Life, Anxiety and Depression in Breast Cancer Survivors: A Systematic Review and Meta-analysis. Asian Nurs Res (Korean Soc Nurs Sci). 2023;17(5):276-85. | Comorbid diseases (cancer), no diagnosed depression according to recognized diagnostic criteria |
| 1. Noetel et al., 2024 | Noetel M, Sanders T, Gallardo-Gómez D, Taylor P, Del Pozo Cruz B, van den Hoek D, et al. Effect of exercise for depression: systematic review and network meta-analysis of randomised controlled trials. Bmj. 2024;384:e075847. | Postpartum depression, comorbid diseases |
| 1. Kvam et al., 2016 | Kvam S, Kleppe CL, Nordhus IH, Hovland A. Exercise as a treatment for depression: A meta-analysis. J Affect Disord. 2016;202:67-86. | Comorbid diseases |
| 1. Bidzan-Wiącek et al., 2024 | Bidzan-Wiącek M, Błażek M, Antosiewicz J. The relationship between physical activity and depressive symptoms in males: A systematic review and meta-analysis. Acta Psychologica. 2024;243:104145. | Cross-sectional studies |
| 1. Rossi et al., 2024 | Rossi FE, Dos Santos GG, Rossi PAQ, Stubbs B, Barreto Schuch F, Neves LM. Strength training has antidepressant effects in people with depression or depressive symptoms but no other severe diseases: A systematic review with meta-analysis. Psychiatry Res. 2024;334:115805. | Several studies included without testing for depression |
| 1. Catalan-Matamoros et al., 2016 | Catalan-Matamoros D, Gomez-Conesa A, Stubbs B, Vancampfort D. Exercise improves depressive symptoms in older adults: An umbrella review of systematic reviews and meta-analyses. Psychiatry Res. 2016;244:202-9. | Sample too old |
| 1. Kim et al., 2023 | Kim M, Lee Y, Kang H. Effects of Exercise on Positive Symptoms, Negative Symptoms, and Depression in Patients with Schizophrenia: A Systematic Review and Meta-Analysis. Int J Environ Res Public Health. 2023;20(4). | Comorbid diseases (Schizophrenia), no diagnosed depression according to recognized diagnostic criteria |
| 1. Gordon et al., 2022 | Gordon BR, McDowell CP, Hallgren M, Meyer JD, Lyons M, Herring MP. Association of Efficacy of Resistance Exercise Training With Depressive Symptoms: Meta-analysis and Meta-regression Analysis of Randomized Clinical Trials. JAMA Psychiatry. 2018;75(6):566-76. | Comorbid diseases |
| 1. Schuch et al., 2018 | Schuch FB, Vancampfort D, Firth J, Rosenbaum S, Ward PB, Silva ES, et al. Physical Activity and Incident Depression: A Meta-Analysis of Prospective Cohort Studies. Am J Psychiatry. 2018;175(7):631-48. | No diagnosed depression according to recognized diagnostic criteria (the study evaluated participants who were free of depression or depressive symptoms at baseline) |
| 1. Feller et al., 2023 | Feller D, Fox I, Gozzer P, Trentin F, Papola D. Exercise for Depressive Symptoms in Parkinson Disease: A Systematic Review and Meta-analysis of Randomized Controlled Trials. Archives of Physical Medicine and Rehabilitation. 2023;104(2):331-9. | Comorbid diseases ( Parkinson Disease), no diagnosed depression according to recognized diagnostic criteria |
| 1. Schuch et al., 2016 | Schuch FB, Vancampfort D, Richards J, Rosenbaum S, Ward PB, Stubbs B. Exercise as a treatment for depression: A meta-analysis adjusting for publication bias. J Psychiatr Res. 2016;77:42-51. | Comorbid diseases |
| 1. Ibeneme et al., 2022 | Ibeneme SC, Uwakwe VC, Myezwa H, Irem FO, Ezenwankwo FE, Ajidahun TA, et al. Impact of exercise training on symptoms of depression, physical activity level and social participation in people living with HIV/AIDS: a systematic review and meta-analysis. BMC Infect Dis. 2022;22(1):469. | Comorbid diseases (HIV/ AIDS), no diagnosed depression according to recognized diagnostic criteria |
| 1. Selzler et al., 2023 | Selzler AM, Ellerton C, Ellerton L, Habash R, Nekolaichuk E, Goldstein R, et al. The Relationship between Physical Activity, Depression and Anxiety in People with COPD: A Systematic Review and Meta-analyses. Copd. 2023;20(1):167-74. | Comorbid diseases (COPD), no diagnosed depression according to recognized diagnostic criteria |
| 1. García-Muñoz et al., 2023 | García-Muñoz C, Villar-Alises O, Rodríguez-Sánchez-Laulhé P, Matias-Soto J, Martinez-Calderon J. No effects were found in favor of Hatha or Iyengar yoga exercises for improving cancer-related fatigue, depression symptoms, or quality of life: a systematic review with meta-analysis and metaregression. Supportive Care in Cancer. 2023;32(1):3. | Comorbid diseases (cancer),  no diagnosed depression according to recognized diagnostic criteria |
| 1. Geng et al., 2023 | Geng L, Duan Y, Li X, Yue S, Li R, Liu H, et al. Comparative efficacy of mind-body exercise for depression in breast cancer survivors: A systematic review and network meta-analysis. Worldviews Evid Based Nurs. 2023;20(6):593-609. | Comorbid diseases (Cancer), no diagnosed depression according to recognized diagnostic criteria |
| 1. R. Chen et al., 2024 | Chen R, Guo Y, Kuang Y, Zhang Q. Effects of home-based exercise interventions on post-stroke depression: A systematic review and network meta-analysis. International Journal of Nursing Studies. 2024;152:104698. | Sample too old |
| 1. Yin et al., 2023 | Yin J, Yue C, Song Z, Sun X, Wen X. The comparative effects of Tai chi versus non-mindful exercise on measures of anxiety, depression and general mental health: A systematic review and meta-analysis. J Affect Disord. 2023;337:202-14. | Comorbid diseases, no diagnosed depression according to recognized diagnostic criteria |
| 1. Wegner et al., 2020 | Wegner M, Amatriain-Fernández S, Kaulitzky A, Murillo-Rodriguez E, Machado S, Budde H. Systematic Review of Meta-Analyses: Exercise Effects on Depression in Children and Adolescents. Front Psychiatry. 2020;11:81. | Sample too old |
| 1. Bourke et al., 2022 | Bourke M, Patten RK, Klamert L, Klepac B, Dash S, Pascoe MC. The acute affective response to physical activity in people with depression: A meta-analysis. Journal of Affective Disorders. 2022;311:353-63. | No comparisons were made with control conditions |
| 1. Jesper et al., 2017 | Jesper K, Carsten H, Helene S, Christian G, Merete N. Exercise for patients with major depression: a systematic review with meta-analysis and trial sequential analysis. BMJ Open. 2017;7(9):e014820. | Comorbid diseases |
| 1. L. Chen et al., 2024 | Chen L, Liu Q, Xu F, Wang F, Luo S, An X, et al. Effect of physical activity on anxiety, depression and obesity index in children and adolescents with obesity: A meta-analysis. J Affect Disord. 2024;354:275-85. | Comorbid diseases, sample too young |
| 1. Piva et al., 2023 | Piva T, Masotti S, Raisi A, Zerbini V, Grazzi G, Mazzoni G, et al. Exercise program for the management of anxiety and depression in adults and elderly subjects: Is it applicable to patients with post-covid-19 condition? A systematic review and meta-analysis. Journal of Affective Disorders. 2023;325:273-81. | Comorbid diseases, no diagnosed depression according to recognized diagnostic criteria |
| 1. Wang et al., 2022 | Wang X, Cai ZD, Jiang WT, Fang YY, Sun WX, Wang X. Systematic review and meta-analysis of the effects of exercise on depression in adolescents. Child Adolesc Psychiatry Ment Health. 2022;16(1):16. | Sample too young |
| 1. Tao et al., 2023 | Tao A, Ho KHM, Yang C, Chan HYL. Effects of non-pharmacological interventions on psychological outcomes among older people with frailty: A systematic review and meta-analysis. International Journal of Nursing Studies. 2023;140:104437. | Sample too old, no diagnosed depression according to recognized diagnostic criteria |
| 1. Rhyner & Watts, 2015 | Rhyner K, Watts A. Exercise and Depressive Symptoms in Older Adults: A Systematic Meta-Analytic Review. Journal of aging and physical activity. 2015;24. | Sample too old, no diagnosed depression according to recognized diagnostic criteria |
| 1. Ji et al., 2024 | Ji M, Li R, Xu Y. Meta-analysis of the effect of different exercise modalities in the prevention and treatment of perinatal depression. J Affect Disord. 2024;350:442-51. | Perinatal depression, no diagnosed depression according to recognized diagnostic criteria |
| 1. Park et al., 2023 | Park S-H, Han K, Jang Y. Effects of Physical Activity on Reducing Depression and Menopausal Symptoms: A Meta-Analysis. Journal of Korean Academy of psychiatric and Mental Health Nursing. 2023;32:325-39. | no diagnosed depression according to recognized diagnostic criteria |
| 1. Xu et al., 2023 | Xu H, Liu R, Wang X, Yang J. Effectiveness of aerobic exercise in the prevention and treatment of postpartum depression: Meta-analysis and network meta-analysis. PLoS One. 2023;18(11):e0287650. | Postpartum depression |
| 1. X. Liu et al., 2023 | Liu X, Wang G, Cao Y. The effectiveness of exercise on global cognitive function, balance, depression symptoms, and sleep quality in patients with mild cognitive impairment: A systematic review and meta-analysis. Geriatr Nurs. 2023;51:182-93. | Cognitive impairment |
| 1. L. Liu et al., 2023 | Liu L, Liu C, Liu X, Yang Y. Summary of the effect of an exercise intervention on antenatal depression and the optimal program: a systematic review and Meta-analysis. BMC Pregnancy Childbirth. 2023;23(1):293. | Postpartum depression |
| 1. Axelsdóttir et al., 2021 | Axelsdóttir B, Biedilae S, Sagatun Å, Nordheim LV, Larun L. Review: Exercise for depression in children and adolescents - a systematic review and meta-analysis. Child Adolesc Ment Health. 2021;26(4):347-56. | Sample too young |
| 1. Luo et al., 2022 | Luo Q, Zhang P, Liu Y, Ma X, Jennings G. Intervention of Physical Activity for University Students with Anxiety and Depression during the COVID-19 Pandemic Prevention and Control Period: A Systematic Review and Meta-Analysis. Int J Environ Res Public Health. 2022;19(22). | No diagnosed depression according to recognized diagnostic criteria |
| 1. Q. Liu et al., 2023 | Liu Q, Ni W, Zhang L, Zhao M, Bai X, Zhang S, et al. Comparative efficacy of various exercise interventions on depression in older adults with mild cognitive impairment: A systematic review and network meta-analysis. Ageing Res Rev. 2023;91:102071. | Cognitive impairment, sample too old |
| 1. Law et al., 2023 | Law CYJ, Yu THJ, Chen T. Effectiveness of aerobic and resistance exercise in cancer survivors with depression: A systematic review and meta-analysis of randomized controlled trials. J Psychosom Res. 2023;173:111470. | Comorbid diseases (cancer), |
| 1. F.B. Li et al., 2023 | Li FB, Lu P, Wu HT, Wang MH, Wang JD. Effects of Music, Massage, Exercise, or Acupuncture in the Treatment of Depression Among College Students: A Network Meta-Analysis. Neuropsychiatr Dis Treat. 2023;19:1725-39. | no diagnosed depression according to recognized diagnostic criteria |
| 1. He et al., 2023 | He L, Soh KL, Huang F, Khaza'ai H, Geok SK, Vorasiha P, et al. The impact of physical activity intervention on perinatal depression: A systematic review and meta-analysis. J Affect Disord. 2023;321:304-19. | Perinatal depression, no diagnosed depression according to recognized diagnostic criteria |
| 1. F. Liu et al., 2020 | Liu F, Cui J, Liu X, Chen KW, Chen X, Li R. The effect of tai chi and Qigong exercise on depression and anxiety of individuals with substance use disorders: a systematic review and meta-analysis. BMC Complement Med Ther. 2020;20(1):161. | Comorbid diseases (substance use disorders) |
| 1. Hooper et al., 2022 | Hooper N, Johnson T, Sachs M, Silverio A, Zhu L, Bhimla A, et al. Comparative Efficacy of Exercise Training and Conventional Psychotherapies for Adult Depression: A Network Meta-Analysis. Commonhealth (Phila). 2022;3(2):47-64. | Comorbid diseases |
| 1. Yan et al., 2021 | Yan LB, Zhang JZ, Zhou Q, Peng FL. Multidimensional analyses of the effect of exercise on women with depression: A meta-analysis. Medicine (Baltimore). 2021;100(33):e26858. | Comorbid diseases |
| 1. Z. Li et al., 2019 | Li Z, Liu S, Wang L, Smith L. Mind-Body Exercise for Anxiety and Depression in COPD Patients: A Systematic Review and Meta-Analysis. Int J Environ Res Public Health. 2019;17(1). | Comorbid diseases (COPD) |
| 1. Salam et al., 2022 | Salam A, Woodman A, Chu A, Al-Jamea LH, Islam M, Sagher M, et al. Effect of post-diagnosis exercise on depression symptoms, physical functioning and mortality in breast cancer survivors: A systematic review and meta-analysis of randomized control trials. Cancer Epidemiol. 2022;77:102111. | Comorbid diseases (cancer), no diagnosed depression according to recognized diagnostic criteria |
| 1. Marconcin et al., 2021 | Marconcin P, Peralta M, Gouveia É R, Ferrari G, Carraça E, Ihle A, et al. Effects of Exercise during Pregnancy on Postpartum Depression: A Systematic Review of Meta-Analyses. Biology (Basel). 2021;10(12). | Pregnancy on postpartum depression, no diagnosed depression according to recognized diagnostic criteria |
| 1. Zhu et al., 2021 | Zhu L, Li L, Li XZ, Wang L. Mind-Body Exercises for PTSD Symptoms, Depression, and Anxiety in Patients With PTSD: A Systematic Review and Meta-Analysis. Front Psychol. 2021;12:738211. | Comorbid diseases (PTBS) |
| 1. Zou et al., 2018 | Zou L, Yeung A, Quan X, Hui SS, Hu X, Chan JSM, et al. Mindfulness-Based Baduanjin Exercise for Depression and Anxiety in People with Physical or Mental Illnesses: A Systematic Review and Meta-Analysis. Int J Environ Res Public Health. 2018;15(2). | Comorbid diseases |
| 1. Nakamura et al., 2019 | Nakamura A, van der Waerden J, Melchior M, Bolze C, El-Khoury F, Pryor L. Physical activity during pregnancy and postpartum depression: Systematic review and meta-analysis. J Affect Disord. 2019;246:29-41. | Pregnancy on postpartum depression |
| 1. Bourbeau et al., 2020 | Bourbeau K, Moriarty T, Ayanniyi A, Zuhl M. The Combined Effect of Exercise and Behavioral Therapy for Depression and Anxiety: Systematic Review and Meta-Analysis. Behav Sci (Basel). 2020;10(7). | Comorbid diseases |
| 1. Miller et al., 2020 | Miller KJ, Gonçalves-Bradley DC, Areerob P, Hennessy D, Mesagno C, Grace F. Comparative effectiveness of three exercise types to treat clinical depression in older adults: A systematic review and network meta-analysis of randomised controlled trials. Ageing Res Rev. 2020;58:100999. | Sample too old |
| 1. Narita et al., 2019 | Narita Z, Inagawa T, Stickley A, Sugawara N. Physical activity for diabetes-related depression: A systematic review and meta-analysis. Journal of Psychiatric Research. 2019;113:100-7. | Comorbid diseases (diabetes) |
| 1. Heinzel et al., 2015 | Heinzel S, Lawrence J, Kallies G, Rapp M, Heissel A. Using Exercise to Fight Depression in Older Adults. GeroPsych: The Journal of Gerontopsychology and Geriatric Psychiatry. 2015;28:149-62. | Sample too old |
| 1. Kelley et al., 2015 | Kelley GA, Kelley KS, Hootman JM. Effects of exercise on depression in adults with arthritis: a systematic review with meta-analysis of randomized controlled trials. Arthritis Res Ther. 2015;17(1):21. | Comorbid diseases, no diagnosed depression according to recognized diagnostic criteria |
| 1. Tu et al., 2014 | Tu R-H, Zeng Z-Y, Zhong G-Q, Wu W-F, Lu Y-J, Bo Z-D, et al. Effects of exercise training on depression in patients with heart failure: a systematic review and meta-analysis of randomized controlled trials. European Journal of Heart Failure. 2014;16(7):749-57. | Comorbid diseases, no diagnosed depression according to recognized diagnostic criteria |
| 1. Adamson et al., 2015 | Adamson BC, Ensari I, Motl RW. Effect of Exercise on Depressive Symptoms in Adults With Neurologic Disorders: A Systematic Review and Meta-Analysis. Archives of Physical Medicine and Rehabilitation. 2015;96(7):1329-38. | Comorbid diseases |
| 1. Poyatos-Léon et al., 2017 | Poyatos-León R, García-Hermoso A, Sanabria-Martínez G, Alvarez-Bueno C, Cavero-Redondo I, Martinez Vizcaino V. Effects of Exercise-Based Interventions on postpartum depression: A Meta-Analysis of Randomised Controlled Trials. Birth. 2017;44. | Pregnant women |
| 1. Pentland et al., 2021 | Pentland V, Spilsbury S, Biswas A, Mottola M, Paplinskie S, Mitchell M. Does Walking Reduce Postpartum Depressive Symptoms? A Systematic Review and Meta-Analysis of Randomized Controlled Trials. Journal of Women's Health. 2021;31. | Postpartum Depression |
| 1. Weinstein et al., 2024 | Weinstein A, Aert R, Donovan K, Muskens L, Kop W. Affective responses to acute exercise: A meta-analysis of the potential beneficial effects of a single bout of exercise on general mood, anxiety, and depressive symptoms. Psychosomatic medicine. 2024. | No diagnosed depression according to recognized diagnostic criteria |
