# Supplementary material for: A 10 years update of effects of exercise on depression disorders—in otherwise healthy adults: A systematic review of meta-analyses and neurobiological mechanisms
Source: PLoS One. 2025 May 5;20(5):e0317610. doi: 10.1371/journal.pone.0317610 (PMC12052119; doi:10.1371/journal.pone.0317610)
Supplement: S2 Table — This table contains a detailed list of all relevant data from the included studies. (DOCX) [file pone.0317610.s004.docx]

| Study | Std. Mean Difference | Lower CI | Upper CI | p | Data extractor | Date of data extraction | Eligibility  Confirmed? |
| --- | --- | --- | --- | --- | --- | --- | --- |
| Bailey et al. [32] | -0.82 | -1.02 | -0.61 | 0.050 | Nina Dolz | from 12.04.2024 to 15.05.2024 | yes |
| Correia et al. [4] | -0.76 | -1.18 | -0.34 | 0.010 | Nina Dolz | from 12.04.2024 to 15.05.2024 | yes |
| Guo et al. [52] | -1.13 | -1.48 | -0.78 | 0.010 | Nina Dolz | from 12.04.2024 to 15.05.2024 | yes |
| Heissel et al. [22] | -0.946 | -1.18 | -0.71 | 0.001 | Nina Dolz | from 12.04.2024 to 15.05.2024 | yes |
| Jazayeri et al. [28] (Resistance) | -0.14 | -0.16 | -0.12 | 0.000 | Nina Dolz | from 12.04.2024 to 15.05.2024 | yes |
| Jazayeri et al. [28] (Endurance) | -0.16 | -0.18 | -0.14 | 0.000 | Nina Dolz | from 12.04.2024 to 15.05.2024 | yes |
| Ju et al. [50] | -0.71 | -0.90 | -0.52 | 0.020 | Nina Dolz | from 12.04.2024 to 15.05.2024 | yes |
| Krogh et al. [48] | -0.66 | -0.86 | -0.46 | 0.001 | Nina Dolz | from 12.04.2024 to 15.05.2024 | yes |
| Lee et al. [51] | -0.62 | -0.86 | -0.37 | 0.000 | Nina Dolz | from 12.04.2024 to 15.05.2024 | yes |
| Morres et al. [7] | -0.79 | -1.01 | -0.57 | 0.000 | Nina Dolz | from 12.04.2024 to 15.05.2024 | yes |
| Pérez-López et al. [49] | -0.36 | -0.52 | -0.21 | 0.002 | Nina Dolz | from 12.04.2024 to 15.05.2024 | yes |
| Recchia et al. [16] | -0.45 | -0.67 | -0.23 | 0.003 | Nina Dolz | from 12.04.2024 to 15.05.2024 | yes |

**S2 Table. Overview of all data extracted from the primary sources for the systematic review and/or meta-analysis.**

**Note. An explanation of how missing data were handled:**

As one of the inclusion criteria was that effect sizes had to be reported, we have no missing data in this regard. If studies did not report effect sizes, these studies were excluded. The original authors’ evaluation and missing risk of bias entries were not replaced by the authors of the present review of meta-analyses.
